# Multiple instance learning of Calmodulin binding sites

**DOI:** 10.1093/bioinformatics/bts416

**Published:** 2012-09-03

**Authors:** Fayyaz ul Amir Afsar Minhas, Asa Ben-Hur

**Affiliations:** Department of Computer Science, Colorado State University, Fort Collins, CO 80523-1873, USA

## Abstract

**Motivation:** Calmodulin (CaM) is a ubiquitously conserved protein that acts as a calcium sensor, and interacts with a large number of proteins. Detection of CaM binding proteins and their interaction sites experimentally requires a significant effort, so accurate methods for their prediction are important.

**Results:** We present a novel algorithm (MI-1 SVM) for binding site prediction and evaluate its performance on a set of CaM-binding proteins extracted from the Calmodulin Target Database. Our approach directly models the problem of binding site prediction as a large-margin classification problem, and is able to take into account uncertainty in binding site location. We show that the proposed algorithm performs better than the standard SVM formulation, and illustrate its ability to recover known CaM binding motifs. A highly accurate cascaded classification approach using the proposed binding site prediction method to predict CaM binding proteins in *Arabidopsis thaliana* is also presented.

**Availability:** Matlab code for training MI-1 SVM and the cascaded classification approach is available on request.

**Contact:** fayyazafsar@gmail.com or asa@cs.colostate.edu

## 1 INTRODUCTION

Calmodulin (CaM) is an intracellular calcium sensor protein that interacts with a large number of proteins to regulate their biological functions and exhibits sequence conservation across all eukaryotes (Bouche *et al.*, 2005). Ca^2+^ plays a very important role in many cellular functions ranging from fertilization and cellular division to neuronal spiking (Reddy *et al.*, 2011). Due to the importance of calcium signaling in cells, identifying proteins that bind CaM and determining the location of the CaM binding site in them can help in gaining a better understanding of cellular function in general, and the role of calcium in different cellular processes in particular. This article presents a highly accurate computational approach that can identify the location of a CaM binding site in a protein solely on the basis of its amino acid sequence, helping avoid the significant effort of performing such experiments in the lab (Reddy *et al.*, 2011). Our approach uses sequence information alone, which ensures its wider applicability in comparison to methods that rely on structural modeling (Zhou and Qin, 2007).

CaM binding sites are known to be contiguous in sequence, often occurring through an amphiphilic alpha helix (O'Neil and DeGrado, 1990). This makes CaM binding site prediction amenable to a sliding-window classification approach, as applied in recent work (Radivojac *et al.*, 2006; Hamilton *et al.*, 2011). The method by Radivojac *et al.* uses a hierarchical neural network classifier trained on the basis of amino acid properties averaged over a fixed-size window. Hamilton *et al.* showed that a simple sliding window Support Vector Machine (SVM) trained on average amino acid composition achieves similar performance.

In this article, we present a novel formulation of the binding site prediction problem that is based on the framework of multiple instance learning (MIL; Dietterich *et al.*, 1997). In MIL positive examples come in bags. For a positive bag, it is assumed that at least one of the examples is indeed positive whereas negative bags contain only negative examples. We use this for binding site prediction by forming a positive bag out of fixed-size sequence windows that overlap the annotated binding site. This allows us to model the uncertainty in actual binding site location–experimental methods may not precisely locate a binding site, and may include a region that is larger than the true binding site due to limitations of budget and experimental procedures. Furthermore, modeling binding sites this way facilitates the use of sequence representations that are position dependent, yielding a more detailed model of the binding site. This allows learning of motifs that are characteristic of the binding site.

MIL has been applied in a variety of other problem domains such as object tracking (Babenko *et al.*, 2011), protein identification (Tao *et al.*, 2004), and prediction of protein–ligand binding affinities (Teramoto and Kashima, 2010).

Our results show that the proposed MI-1 SVM has higher accuracy than the classical multiple instance SVM (Andrews *et al.*, 2003), and is also faster to train. MI-1 also performs better than a standard SVM, thereby improving on existing work of (Radivojac *et al.*, 2006; Hamilton *et al.*, 2011). We also compare the merits of several ways of representing binding sites, and demonstrate the ability of our method to learn motifs that are associated with CaM binding. Finally, we show how the resulting binding site predictor can be used as the basis for a classifier that predicts CaM binding proteins, with improved accuracy over earlier work.

## 2 METHODS

### 2.1 Datasets and pre-processing

The dataset for CaM binding site prediction and its pre-processing follows (Radivojac *et al.*, 2006). A set of 210 proteins was obtained from the Calmodulin Target database (Yap *et al.*, 2000). Each of these proteins bind CaM, and one or more binding sites within each protein are annotated. A non-redundant subset of 153 proteins containing 185 binding sites was then chosen such that no two proteins have more than 40% sequence identity and no two binding sites are more than 50% identical.

Sequence windows of length 21, the average length of CaM binding sites, were extracted from the protein sequences to create positive and negative examples. Negative examples were created by sliding a length 21 window in 10 amino acid increments such that no part of the window overlaps an annotated binding site. Positive examples, on the other hand, were created by sliding a length 21 window over an annotated binding site in increments of 1 amino acid. Thus, the number of positive examples from an annotated binding site equals the number of amino acids in the binding site.

For CaM binding prediction, we used a dataset of 236 proteins experimentally determined to bind CaM using a protein array screen that tested around a thousand proteins in *Arabidopsis thaliana* (Popescu, 2007). The remaining 27,140 proteins in the *A. thaliana* proteome were used as negative examples (non-binders).

### 2.2 Vanilla SVM

As a baseline method we have used a standard binary SVM (Cortes and Vapnik, 1995). Our labeled dataset consists of *N* labeled examples (*x_i_,y_i_*) where *x_i_*is the sequence of a window, and *y_i_ ∈* {+1,–1} is its associated label indicating whether the central residue of *x_i_* lies in a binding site or not. The large-margin learning problem can be formulated as:
(1)
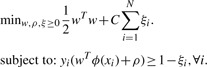


Here *ϕ* (*x_i_*) is the feature representation of the window *x_i_* and the cost parameter *C* controls the trade-off between constraint violation and margin maximization. The discriminant function *f* (*x_i_*) = *w^T^ ϕ* (*x_i_*) + *ρ* can then be used to predict whether a given window is part of a binding site or not. The location of a binding site is predicted by the window that offers the highest value of the discriminant function for that protein (Hamilton *et al.*, 2011). PyML (Ben-Hur, PyML—machine learning in Python, 2011) was used for the implementation.

### 2.3 Multiple instance learning SVM

In MIL [both multiple instance learning SVM (mi-SVM) and MI-1-SVM], the positive examples from each binding site are grouped into a single bag. We denote the set of positive examples for a given binding site *b* as *P*(*b*) and the set of negative examples from the protein to which the binding site *b* belongs as *N* (*b*). The mi-SVM approach is formulated as follows (Andrews *et al.*, 2003):
(2)
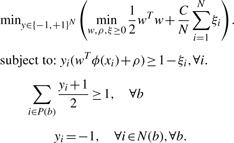


In this formulation *y_i_*∈ {+1,–1} acts as a label for the window *x_i_*, and the objective is to find the optimal labeling of the examples that comprise the positive bags such that at least one example in each positive bag is labeled as positive 
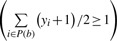
. The other constraints ensure correct labeling of the given training examples and that all negative examples are labeled as negative examples. In case of the binding site prediction problem, this means that a trained mi-SVM will choose at least one positive window from the set of positive windows in a binding site. The mi-SVM formulation is a combinatorial optimization problem. We use the heuristic algorithm proposed by (Andrews *et al.*, 2003) to solve this problem. The algorithm initially assigns the label of a bag to all examples in it, i.e. all examples in positive bags are assigned a label of +1 whereas all negative examples are assigned –1. It uses these assigned labels to solve a regular SVM learning problem [as in Equation (1)]. Labels for all examples in positive bags are then imputed based upon the sign of their discriminant function value. If no example in a positive bag is assigned a positive label (i.e. the constraint 
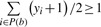
 is violated), the algorithm picks the example in the bag having the largest discriminant function value and sets its label to +1. The algorithm then alternates between label imputation and SVM training until the labels stop changing. This simple algorithm has shown good performance in comparison to more complicated ones (Andrews *et al.*, 2003).

### 2.4 Novel MI SVM formulation (MI-1 SVM)

**Fig. 1.**
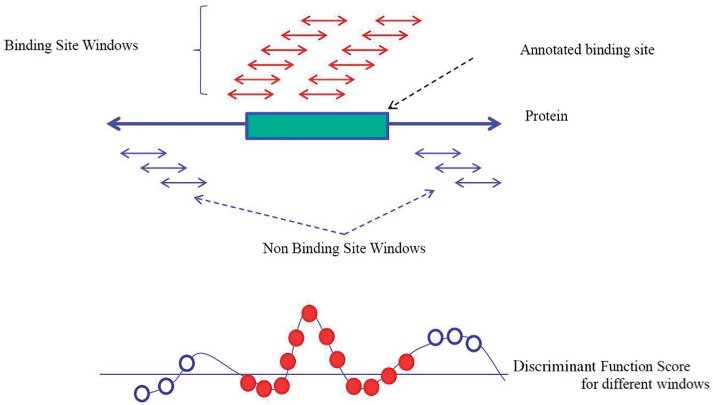
CaM binding site prediction with MIL. The annotated binding site is shown as a box, and is represented by a ‘bag’ composed of the windows indicated in red above the binding site. The rest of the windows that do not overlap the binding site are negative examples (shown in blue below the protein). The bottom panel illustrates the desired characteristics of the classifier's discriminant function. The dots indicate the score of different examples (positive indicated by solid red circles and negative shown as hollowed blue circles). The score from the trained discriminant function for one window in a binding site should be higher than the scores generated for non-binding site windows within that protein

Accurate prediction of the location of a binding site in a protein requires a less stringent condition than the one used in mi-SVM: at least one window in the true binding site needs to score higher than the negative windows *from the same protein* (Fig. 1). This allows us to significantly reduce the complexity of the learning problem in comparison to mi-SVM. The mi-SVM and vanilla SVM formulations try to classify windows as binding or non-binding without modeling the concept that these windows in fact lie within a protein. Our proposed MI-1 SVM formulation, on the other hand, operates at the protein level. The large-margin formulation of this learning problem, can be expressed as follows:
(3)
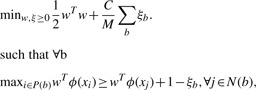

where *M* is the total number of binding sites in the training data. For a given binding site, this formulation tries to maximize the difference between the discriminant function values of the maximum scoring window within the binding site and the non-binding windows in the rest of the protein containing that binding site. Since MI-1 SVM simply compares the discriminant function scores in the binding and non-binding site windows in its constraints, it does not require a bias term. Moreover, the number of slack variables (*ξ_b_*) in MI-1 SVM is equal to the number of binding sites and not the number of training examples, as in the vanilla SVM and the mi-SVM. As a consequence, the number of variables involved in the optimization in MI-1 SVM is much smaller than that in mi-SVM and this leads to faster training. Using the same *ξ_b_* for a single binding site effectively takes the maximum of the scores over all non-binding site windows of the protein to which *b* belongs. Another important feature of MI-1 SVM is that, like the ranking SVM discussed in (Joachims, 2006), MI-1 SVM also explicitly maximizes the area under the Receiver Operating Characteristic (ROC) curve.

Similar to mi-SVM, which performs optimization over the labels of examples in positive bags, MI-1 SVM is also a combinatorial optimization problem because of the maximum operation in its constraints. We have used the heuristic algorithm given in Table 1 to obtain a solution to this problem. The algorithm can be stopped when the representative examples of all binding sites stop changing, or on the basis of a user-defined maximum number of iterations. In all our experiments, the algorithm converged in 10 iterations or less. A trained MI-1 SVM can be used to produce discriminant function scores for any given residue in a protein.

**Table 1.** Heuristic algorithm used for training MI-1

*Initialization*:
With each binding site *b*, we associate a representative example *x*^b^ with feature representation *ϕ*(*x*^b^) which is initialized to be the mean of the examples in *P*(*b*):
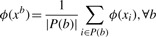
*Until convergence, repeat*:
Solve the following quadratic programming (QP) problem:
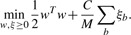
such that, ∀ *b*
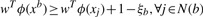
Update (for all binding sites):
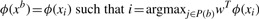

The QP problem in the MI-1 algorithm can be solved in the primal or in the dual. The primal formulation of the problem (3) is more efficient than the dual when the dimensionality of the feature vector is smaller than the number of training examples. The dual formulation of the QP problem (based upon the Lagrange of the primal) is given by:
(4)
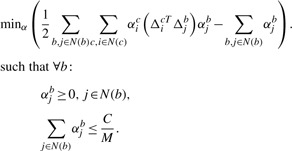


Here *α_j_^b^* is the Lagrange variable corresponding to the primal constraint *w^T^ϕ*(*x^b^*) ≥ *w^T^ ϕ*(*x_j_*) +1–ξ_*b*_ and Δ*_j_^b^* = *ϕ*(*x^b^*)–*ϕ*(*x_j_*). The dual formulation reveals some interesting aspects of the MI-1 SVM. It shows that Lagrange variables (*α*) only exist for negative examples, and that the sum of all *α* for negative examples from a single protein is constrained to be less than or equal to *C/M*. This differs from a conventional SVM formulation which requires that each of the *α*, on its own, should be ≤ *C/M* and the sum of products of *α* from all training examples with their corresponding labels should be zero. Thus, the MI-1 SVM formulation is less constrained than a conventional SVM formulation and this can potentially lead to a better solution.

### 2.5 CaM binding prediction

In this article, we compare the following two strategies for CaM binding prediction.

#### 2.5.1 Discriminant function scoring

The maximum discriminant function score across all windows in a protein can be used as the CaM binding propensity of that protein. This approach was used in (Hamilton *et al.*, 2011) to predict CaM binding of proteins in the *A. thaliana* proteome. In their method, the scores were generated using a standard SVM classifier trained for binding site prediction. In this article, we use the scores from MI-1 SVM instead.

#### 2.5.2 Cascaded classification

We implemented a two-stage cascaded classification approach for CaM binding prediction. In the first stage, the window in a given protein with the highest MI-1 SVM discriminant function score is chosen as the most likely binding site window for that protein. This is done for all proteins in the training set. In the second stage, a standard SVM is trained to discriminate between the most likely binding site windows in positive examples (known CaM-binding proteins) and negative examples (non-CaM-binding proteins). Once the second stage SVM has been trained, the binding propensity of a test protein can be estimated by first finding its most likely binding site window using MI-1 SVM, and then evaluating the discriminant function value of the second stage SVM for the chosen window. AGaussian kernel was used in the second stage SVM as it performed significantly better than a linear kernel. However, the use of non-linear kernels in MI-1 SVM did not seem to improve performance.

### 2.6 Feature representations

The performance of the learning methods described above for binding site and CaM binding prediction was analyzed using a number of feature representations which are presented next.

#### 2.6.1 p-Spectrum

The *p*-*s*pectrum *ϕ*(*x*) of a string over an alphabet Σ is a vector each of whose components *ϕ_v_*(*x*) are the number of occurrences of each length-*p* substring *v* in the string *x*. The *p*-spectrum kernel between two strings is given by the corresponding Euclidean dot product (Leslie *et al.*, 2002).

#### 2.6.2 Position-dependent p-spectrum

The position-dependent *p*-*s*pectrum *ϕ*(*x*) of a string *x* is a vector of indicator variables *ϕ*_(*v,k*)_(*x*) each showing whether the length-*p* substring *v* occurs at position *k* in the string *x*. The resulting position-dependent *p*-spectrum kernel is given by: *K^PD^* (*x,x*′) = *ϕ*(*x*)^*T*^ *ϕ*(*x*′). The position-dependent kernel takes the relative position of an amino acid in a window into account whereas the *p*-spectrum kernel does not.

#### 2.6.3 Position-dependent gappy triplet

This feature representation quantifies the occurrences of motifs of the form *a*x*^m^b*x*^n^c*, where *a*, *b*, *c* are amino acids and x^*m*^ indicates *m* don’t-care positions. For a given string *x*, the feature vector *ϕ^m,n^*(*x*) of the position-dependent gappy triplet comprises of variables *ϕ*_(*a,b,c,k*)_^*m,n*^ (*x*) which indicate whether the motif *a*x*^m^b*x*^n^c* starts at position *k* in the string or not. The kernel *K^m,n^* (*x,x′*) = *ϕ^m,n^*(*x*)^*T*^ *ϕ^m,n^* (*x′*) between two strings tells us the number of locations in the two strings that have the same motif starting at them. We have used multiple position-dependent gappy triplet kernels as 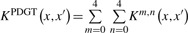. This kernel allows us to extract meaningful information about motifs for CaM binding sites and is only used for binding site prediction for this purpose.

We perform normalization of any kernel representation using the cosine Kernel 
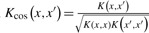


### 2.7 Evaluation methodology

We use Leave-One-Protein-Out (LOPO) cross-validation in order to analyze the performance for binding site prediction. In LOPO, all examples (positive or negative) from a single protein are held-out while the classifier is trained on the remaining proteins. The classifier is then evaluated over the examples from the held out protein. We evaluate the following performance metrics and use their average across all proteins to make comparisons between methods and kernels:

- Protein level area under the ROC curve (AUC): The area (expressed as percentage) under the Receiver Operating Characteristic (ROC) curve (the plot of true positive rate versus false positive rate) obtained for windows in a given protein.
- Protein level area under ROC 10% curve (AUC_0.1_): The area (expressed as percentage) under the ROC curve based on up to the first 10% false positives in a protein.
- False-Hit ratio (FH-measure): The percentage of non-binding site windows (out of the total number of non-binding site windows) that have a score higher than the maximum scoring window in the known binding site. This measure tells us how many non-binding site windows are expected with a score higher than the true binding site window.
- True-Hit probability (TH-measure): For a given protein, a true hit is defined to occur when the residue at the center of the highest scoring window for that protein lies within a binding site. The average number of true hits across all proteins (called the TH-measure) represents the probability of the maximum scoring window predicted by a classifier to lie within a true binding site.

The AUC is a measure of how good a particular method is in ranking binding site windows above non-binding sites. AUC_0.1_ gives us a sense of how good are the top scoring windows produced by a classifier. The FH measure represents the chances of a non-binding site window to be ranked higher than a true binding site window. The TH-measure tells us about the chances of the highest scoring window predicted by a classifier to belong to a true binding site. Both the TH and the FH measures provide meaningful information about the accuracy of the method to a biologist who intends to use the proposed prediction scheme to verify potential binding site locations experimentally.

We use AUC as the performance metric for CaM binding prediction. AUC can be directly computed from the estimated CaM binding propensities when using the Discriminant function scoring approach. With the Cascaded classification approach, AUC is obtained from 5-fold stratified cross-validation with nested grid search for model selection. In cross-validation, it was ascertained that two proteins with more than 40% sequence similarity are in the same fold (evaluated using BLASTCLUST from the NCBI BLAST package (Altschul *et al.*, 1990)). Moreover, the data for CaM binding prediction in *A. thaliana* did not include any proteins which were part of the MI-1 training set.

### 2.8 Model selection

In order to perform model selection (the choice of the cost parameter *C*) for the vanilla and MI-1 SVM formulations for binding site prediction, we used nested 5-fold cross-validation within each iteration of LOPO cross-validation. The TH-measure obtained from the 5-fold cross-validation is then used to choose the value of *C* for that iteration of LOPO. The values of *C* that were used in the nested cross-validation are {0.01, 0.1, 1.0, 10, 100}.

As mi-SVM takes a long time to train, nested cross-validation could not be performed. Instead we evaluated the LOPO cross-validation performance (TH-measure) of mi-SVM with different values of *C* in {0.01, 0.1, 1.0, 10, 100} and the best results with the optimal value of *C* = 10 are reported. This method for selection of *C* for mi-SVM can potentially lead to over optimistic performance estimates. This is not an issue, since our claim is that the proposed approach performs better.

In the case of CaM binding prediction in *A. thaliana* using cascaded classification, we performed a nested (5-fold) grid search within each cross-validation fold for selecting the parameter values of the second-stage SVM. Values of C in the SVM and γ of the Gaussian kernel (*K*(*x*_1_, *x*_2_) = exp (−*γ*|| ϕ(*x*_1_)–ϕ(*x*_2_)||^2^)) were chosen from {0.1, 1, 10, 100} and {0.005, 0.02, 0.5, 2.0}, respectively.

## 3 RESULTS AND DISCUSSION

Table 2 presents the LOPO cross-validation results for the three SVM formulations for the 1-spectrum, position-dependent 1-spectrum and the combination of the two feature representations for predicting CaM binding sites. We observe that both MIL formulations (mi-SVM and MI-1 SVM) perform better than the vanilla SVM. This shows the value of expressing binding site prediction as an MIL problem. This is particularly evident with the use of position-dependent feature representations, as they are more sensitive to changes in relative position of an amino acid in a window within the binding site than position-independent feature representations. It can also be noted that the accuracy of MI-1 SVM is noticeably better than mi-SVM. We believe that this improvement stems from the fact that the proposed scheme implements a more realistic model of the binding site prediction problem. The improvement resulting from switching to a position-dependent feature representation is also larger for MI-1 SVM than that observed in the case of mi-SVM. The higher AUC_0.1_ scores indicate the improved sensitivity and specificity of MI-1 SVM which is also reflected in the ~8% improvement in the TH-measures and the decrease in the FH-measure.

**Table 2.** Results across methods and kernels

Method	Features	AUC	AUC_0.1_	TH %	FH %
Vanilla SVM	1-Spec	95.5	53.9	**66**	2.6
	PD-1	95.6	54.5	64	2.5
	Comb.	95.9	55.1	65	2.1
	*Max. Std.*	0.16	0.59	2.2	0.15
mi-SVM	1-Spec	95.5	**54.4**	64	2.6
	PD-1	96.0	55.8	69	2.1
	Comb.	96.2	55.6	68	1.9
MI-1 SVM	1-Spec	**96.0**	54.3	62	**2.1**
	PD-1	**96.8**	**58.5**	**72**	**1.3**
	Comb.	**96.9**	**59.0**	**75**	**1.2**
	*Max Std.*	0.14	0.80	3.4	0.11
	*Gappy*	96.5	58.5	68	1.6

The features are 1-spectrum (1-Spec), position-dependent 1-spectrum (PD-1) and the combination (Comb) of the 1-Spec and PD-1 representations. The Max Std. rows show the maximum standard deviation of a particular performance metric using the above feature representations. Results with the position-dependent Gappy triplet kernel (Gappy) with MI-1 SVM are also reported (for a single run due to its longer computational time). Bold numbers indicate the best value (across all methods) for a particular metric using a particular feature representation. (AUC: area under the ROC curve, AUC_0.1_ AUC for first 10% false positives, TH: true hit, FH: false hit).

The vanilla SVM approach is the same as the method in (Hamilton *et al.*, 2011), which they showed works comparably as the neural network approach of (Radivojac *et al.*, 2006). Therefore we conclude that the proposed scheme performs better than previously reported approaches.

We also compare the performance of these approaches with a naive local alignment-based method for finding CaM binding sites. In this method, local alignment between a held out protein and the binding sites of the remaining proteins is performed and if the best scoring alignment overlaps (by at least 10 residues) with the known binding site in the held out protein, it is considered to be a true hit. This approach gives a TH% of 39.5%. This shows that the machine learning approaches presented in this article use more than sequence similarity to make better predictions.

We have also performed an analysis of the stability of the results for the MI-1 and the vanilla SVMs by averaging performance statistics of 12 runs of 5-fold cross-validation. This analysis was not performed for mi-SVM or for the gappy triplet kernel with MI-1 SVM owing to their large time requirements. The 5-fold cross-validation results for both the methods are very similar to the LOPO cross-validation results. The maximum standard deviation in a particular performance metric across different feature representations obtained from the 5-fold cross-validation for vanilla and MI-1 SVMs is given in Table 2. This statistic gives an idea of the variability of the results with respect to changes in the data.

**Fig. 2.**
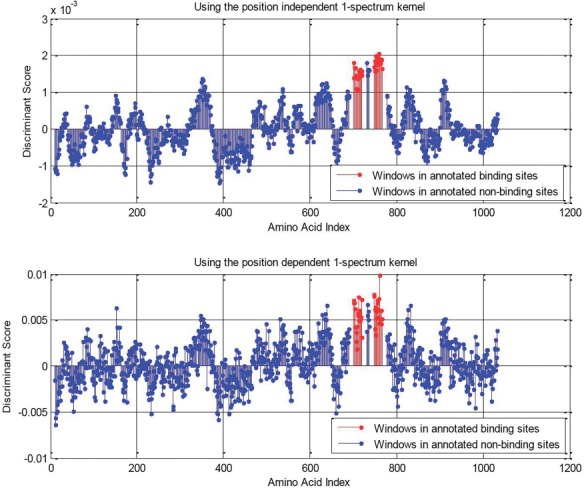
MI-1 discriminant values along the length of a held-out protein with the position-independent (top) and the position-dependent (bottom) 1-spectrum features

Figure 2 shows the output of the MI-1 SVM for a single protein for the position-dependent and position-independent versions of the 1-spectrum feature representation. It is quite clear that the output for the position-independent features is much smoother than that from the position-dependent 1-spectrum features. This is because the position-independent 1-spectrum feature vector representation changes only slightly as the window is translated by one position, whereas the position-dependent feature vector can change dramatically. Due to the increased resolution power, the position-dependent features lead to a classifier that is able to correctly predict both binding sites in the example shown in Figure 2, which is not achieved using the position-dependent features.

**Fig. 3.**
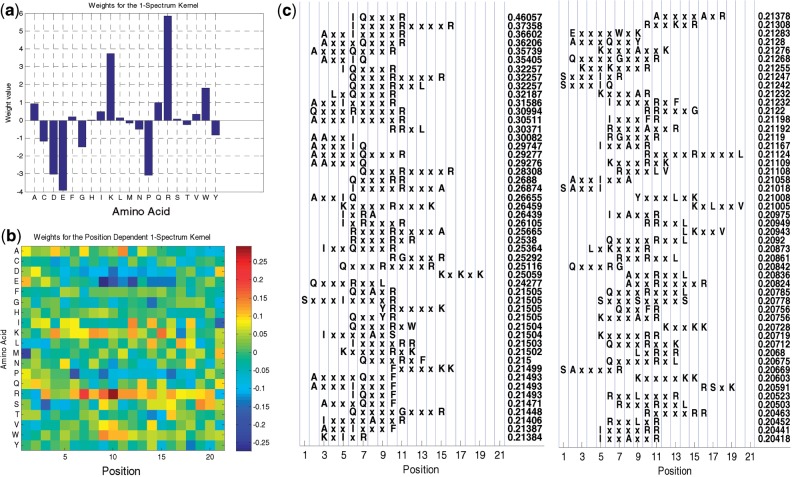
(**a**) Weights of different amino acids in the (position-independent) 1-spectrum feature representation; (**b**) Heat map of the weights of different amino acids versus their position from the MI-1 SVM position-dependent 1-spectrum feature representation; and (**c**) Top 100 (in terms of their weights) motifs from the position-dependent gappy triplet kernel. The last (numeric) column shows actual weight values

We have also analyzed the weight vectors from different feature representations in order to extract amino acid patterns informative of CaM binding sites. The plot of weights from the 1-spectrum features and the position-dependent 1-spectrum features are shown in Figure 3a and b, respectively. The weights for the 1-spectrum features closely follow the amino acid propensities in CaM bindings sites (Hamilton *et al.*, 2011), with R (Arginine), K (Lysine) and W (Tryptophan) showing large positive weights, whereas D (Aspartic acid), E (Glutamic acid) and P (Proline) have large negative weights. The plot of the position-dependent 1-spectrum features indicates that the importance of different amino acids varies with their position in the window. For example, Arginine shows large positive weights in the middle of the window, and negative weights in the ends; Glutamic acid shows the opposite behavior. This indicates that the classifier is indeed learning a position-dependent model.

The results of 5-fold cross-validation using the position-dependent gappy triplet kernel (*K*^PDGT^) shown in Table 2 indicate that this kernel provides comparable performance to other feature representations using MI-1 SVM. Since the number of dimensions in the feature representation of the gappy triplet kernel is much larger than the number of training examples, MI-1 SVM learning was performed using the dual formulation for this kernel, which is more computationally intensive. That is why we have used 5-fold cross-validation instead of LOPO cross-validation.

Next, we ranked the features of the gappy triplet kernel in terms of their weights in MI-1 SVM learning in order to find motifs that are associated with CaM binding. Figure 3c shows the top 100 motifs and their positions. We observe that motifs tend to associate with particular positions, showing that MI-1 SVM uses the flexibility in choosing a representative window to ‘align’ instances of CaM binding sites (for instance, notice the presence of ‘R’ at positions 10 and 11 across different features). Moreover, it is able to find parts of known CaM binding motifs provided in the CaM Target Database (Yap *et al.*, 2000). The CaM Target Database classifies CaM binding targets into 5 groups, each characterized by certain motifs: 3 predominantly calcium-dependent motifs (1–10, 1–14 and 1–16, named according to the position of large hydrophobic residues), the IQ motif which is typically not dependent on calcium concentration, and others. As is evident from Figure 3c, IQ, QxxxR, RxxxxR, RGxxxR, RxxL, KxxxxR receive large positive weights. These motifs are components of the IQ subclass of motifs. Other features belonging to different subclasses of motifs that receive large positive weights include: AxxI, IxxxF, LxxV, (from the 1–14 subclass), RR, KK, RxF (from the 1–10 subclass) etc. This clearly illustrates the capabilities of the proposed scheme to learn CaM binding motifs. We also note that most of the top ranking features correspond to a motif with 3 or 4 do not care positions. This is in agreement with the known fact that CaM binding usually occurs via an alpha helix, and this corresponds to the periodicity of the alpha helix.

On the task of CaM binding prediction (Table 3), the performance of discriminant function scoring is only marginally better than that of the 1-spectrum feature representation used in (Hamilton *et al.*, 2011). However, with the cascaded classification approach with a Gaussian kernel, the results are significantly better. Even though the AUC for the position-independent 1-spectrum features is higher than that of the position-dependent features, the AUC_0.1_ was higher for position-dependent features (29.1) in comparison to the simple 1-spectrum features (26.6).

**Table 3.** Results of CaM binding prediction for Discriminant function scoring and Cascaded classification with an SVM with a Gaussian kernel

Method	Features	AUC
Discriminant function scoring	1-Spec	71.9
	PD-1	70.1
	Comb.	71.9
Cascaded classification	1-Spec	**75.3**
	PD-1	**71.1**
	Comb.	**72.3**

The features are 1-spectrum (1-Spec), position-dependent 1-spectrum (PD-1) and the combination (Comb) of the 1-Spec and PD-1 feature representations. Using Cascaded Classification with a liner kernel in the second stage SVM instead of the Gaussian kernel, the best AUC was 0.72 with 1-spectrum features. (AUC: area under the ROC curve).

In order to obtain a better understanding of what our classifier picks up, we considered the proteins that are not known to bind to CaM and ranked that list according to the score provided by our classifier. We then tested for enrichment of GO terms of segments of that list: the first 1000 proteins, proteins 1001–2000 etc., using the GOrilla tool (Eden *et al.*, 2009). For the first 1000 we found enriched terms that are in agreement with known functions of CaM binders (Reddy *et al.*, 2011): In GO molecular function, transcription function activity and CaM-dependent kinase activities were the most highly enriched with adjusted *p*-values below 10^−10^. All other enriched terms were related to these except for ‘inward rectifier potassium channel activity’ which had an adjusted *p*-value of 0.02. In GO biological process namespace all the terms except for ‘response to carbohydrate stimulus’ (adjusted *p*-value 0.02) were related to phosphorylation and various regulatory processes. In analyzing enrichment for size-1000 chunks we found that the *p*-values for these functions and processes went down as we went down the ranked list, and for proteins ranked 5000–6000, no terms showed enrichment.

## 4 CONCLUSIONS AND FUTURE WORK

We have presented a novel MIL algorithm for CaM binding site prediction called MI-1 SVM, and shown its performance advantages in comparison to the standard MIL SVM and regular SVM, which was used in previous work. Our new MIL formulation captures the minimal constraints that a good binding site classifier needs to have, and we believe this is the reason for its better accuracy. Not only that, it also runs more than twice as fast as standard MIL SVM (running time on a dataset of 16,060 windows was 510.5s for MI-1, 1059.1s for mi-SVM, and 348.3s for vanilla SVM).

Expressing binding site prediction as an MIL problem is a natural way to incorporate uncertainty about binding site location, and our results show that this allows the classifier to ‘align’ binding sites and learn position-dependent motifs that characterize the binding site. The proposed scheme also shows its efficacy in prediction of CaM binding proteins.

In general, binding sites in proteins nucleic acids are not contiguous in sequence as they are in CaM binding proteins. MI-1 SVM can be extended to solve the generic problem of binding site prediction by using sequence-based features that capture the non-contiguous nature of binding sites. Currently, MI-1 SVM generates the CaM binding propensity along a protein's length and cannot explicitly identify multiple binding sites. Identifying the number of binding sites in a protein remains for future work.
